# Ailing Hearts and Troubled Minds: An Historical and Narratological Study on Illness Narratives by Physicians with Cardiac Disease

**DOI:** 10.1007/s10912-020-09610-0

**Published:** 2020-01-28

**Authors:** Jonatan Wistrand

**Affiliations:** Faculty of Medicine, Department of Medical History, Lund University, Jan Waldenströms gata 15, 205 02 Malmö, Sweden

**Keywords:** Physician narratives, Literature and medicine, Cultural history, Sociology

## Abstract

A number of studies show that when doctors become ill, there is often ambiguity in the division of roles and responsibilities in the medical encounter. Yet little is known about how the dilemma of the sick doctor has changed over time. This article explores the experience of illness among physicians by applying an historical, narratological approach to three doctor’s narratives about personal cases of cardiac disease: Max Pinner’s from the 1940s, Robert Seaver’s from the 1980s, and John Mulligan’s from 2015. Drawing on Erving Goffman’s principles of social interaction, I argue that part of the challenge in the analysed narratives is because when doctors seek medical attention for themselves, the ensuing medical ‘drama’ suffers. I compare the three narratives to argue that the experience of becoming a patient while simultaneously remaining a doctor is a challenge that has changed over time. In Pinner’s narrative, the patient identity is both undesirable and inaccessible; in Seaver’s, role ambivalence between doctor and patient is the most salient feature; for Mulligan, his personal rather than professional experience of illness is the overarching theme of the narrative. Finally, I suggest that an awareness of how the medical drama often changes when doctors are patients might prove beneficial both for the doctor-patients and providers of medical care.

## Introduction

When physicians become ill, their professional training is not only a resource but also an impediment (McKevitt and Morgan 1997). Articles based on questionnaires and interviews with practising physicians have disclosed a precarious culture of informal consultations and extensive self-medication, resulting in diffused responsibility, delayed diagnosis, and an overall risk of impaired treatment (Campbell and Delva 2003; Kay et al. 2008; Khalaila, Margolin, and Peleg 2016). But, while in recent decades the dilemma of the sick doctor has attracted increasing academic interest, only modest scholarly attention has so far been paid to how this dilemma has changed over time (Marchalik 2019).

In this essay, I explore the physician’s personal experience of cardiac illness by applying a comparative and narratological approach to written illness narratives, drawing on the testimonies of the pulmonologist Max Pinner in the 1940s, the gastroenterologist Robert Seaver in the 1980s, and the emergency medicine physician John Mulligan in 2015 (Pinner 1952; Seaver 1987; Mulligan 2015). The aim of the study is dual. First, the three doctors’ personal narratives are used to nuance our understanding of the complex set of considerations that guide doctors when they fall ill. For since written testimonies, unlike questionnaires and interviews, are not bound to fit a certain template, they provide complementary insights into illness experience and help-seeking behaviour on the part of physicians. Following Erving Goffman’s principles of social interaction, I argue that at least some of the challenges described in the analysed narratives are due to the fact that, whenever doctors seek health care for themselves, the unfolding medical drama is disorganised (Goffman 1959). The second aim of the article is to investigate how the sick doctor’s experience has changed over time. I compare the narratives and argue that different decades of the twentieth and twenty-first centuries can broaden our understanding of how medical practitioners shaped their experience of personal illness at various stages of the evolution of modern medicine and how this experience interacted with their professionally modelled identities and understandings of disease.

The decision to limit the analysis to narratives about cardiac illness calls for explanation. Compared to many other diagnoses, heart disease carries an existential dimension which for most people, quite apart from any medical and/or surgical interventions, also gives rise to profound reflection. Moreover, from a sociological point of view, cardiology has an interesting history. Until at least the mid-twentieth century, coronary sclerosis was, in the academic literature, described as a ‘doctor’s disease,’ with higher presumed prevalence rates among physicians than any other profession (Levine and Hindle 1945). Whether this notion had any influence on how physicians who had heart disease related to their symptoms is an interesting question that bears investigation.

## Discrepant roles, Erving Goffman, and the disorganisation of the medical drama

A valuable, though underexplored, source for how medical practitioners relate to their own ill health is the growing number of pathographies—autobiographies about illness experience—written by doctors. In accounts such as the neurologist Oliver Sacks’s *A Leg to Stand On* (1984), the dermatologist David Biro’s *One Hundred Days* (2001), and, more recently, the neurosurgeon Paul Kalanithi’s *When Breath Becomes Air* (2016), thoroughly self-reflective reasoning on the quandary of the sick doctor are unveiled.^1^ One common denominator in these literary testimonies is a sense of uncertainty about how to combine their identity as a patient with their professional role as a doctor (Hahn 1985). Since physical wellbeing and mental endurance are regarded as vital attributes of a good doctor, illness and sick leave carry a sense of guilt and stigma for the doctors affected (Ingstad and Moe Christie 2001).^2^ When physicians find themselves ‘on the other side of the doctor’s desk,’ their accustomed role performance is blurred, and faced with a medical setting that is at once familiar and distorted, their understanding of how to act is confused (Mandell and Spiro 1987). In that sense, the experience described in these narratives resembles what sociologist Erving Goffman has termed a ‘disorganised drama’ (1959).

According to Goffman, various contexts of social interaction, including hospitals, can best be understood using the principles of dramaturgy. Much like a play, such milieus are defined by a set of rules and a number of roles, creating predictability in what to expect from one another. As a consequence, Goffman argues, dramas unfold where the actors involved—doctors and patients—have a sense of the script to follow and how to interact with one another.^3^ However, Goffman notes, ‘a performance is a delicate fragile thing that can be shattered by very minor mishaps’ (56). One such difficulty is when individuals who would normally be teammates find themselves in ‘relations to the team which are not apparent and which complicate the problem of putting on a show’ (239): theirs is thus a ‘discrepant role,’ and the social ordering becomes indistinct and disorganised. A central mechanism in this dramaturgical disorganisation is the blurring of the distinction between a back region ‘where the performance of a routine is prepared’ and a front region ‘where the performance is presented’ (238). According to Goffman, those who acquire a discrepant role often possess knowledge of the back region, which reduces their ability to conform to the expected rules of the drama. Though formulated six decades ago, Goffman’s theory of discrepant roles and separate regions still offers a fruitful model when investigating the difficulties experienced by ‘doctor-patients.’ When applied to the narratives I analyze in this article, it is clear that part of the dilemma described by doctor-patients seems to stem from their inability to find a natural place in a medical drama, because their accustomed pattern of behaviour has been deranged by disease.

## A brief social history of cardiac illness

In poetry and fiction, there are innumerable examples of broken hearts representing grief and misfortune. Historically speaking, though, the correlation between mental distress and heart disorder was not just a poetic metaphor but a medical reality. When in 1910 Sir William Osler described angina pectoris in the *Lancet* as ‘a *morbus medicorum*, a disease to which doctors were prone, the principal aetiology Osler suggests was the exceptional ‘worries’ that doctors experience at work. As doctors approached their sixties, Osler concluded, it was imperative that they ease their workload in order to avoid cardiac disease. And this notion of angina pectoris as a somatic disease with emotional roots, particularly likely to afflict doctors, held good until the mid-twentieth century. In an article in *JAMA* in 1937, the cardiologist Harry L. Smith concluded that the incidence of coronary sclerosis was ‘highest of all among physicians.’ Smith shared Osler’s view that the reason for the high levels of heart disease among doctors was mental rather than physical, caused by a combination of ‘stress, strain, intensity of work and mental worries’ (Smith 1937, 1329). The assumption that angina pectoris was a doctors’ disease gave it a certain romantic lustre and attenuating epithets such as ‘disease of the intelligentsia’ and ‘evidence of eminence’ (1327). However, when in the second half of the twentieth century the impact of smoking, diabetes, physical inactivity, and obesity replaced mental stress as the major pathogenesis of coronary sclerosis, the cultural connotations of heart disease also changed. Advances in medical science and technology meant that cardiovascular ailments were increasingly treatable with surgical procedures and pharmaceutical interventions. Oldfield and Jones (2014) have shown how American fiction about individuals with angina pectoris falls into two distinct eras: narratives before the 1960s and those after. While stories in the earlier group revolve around the difficulties of adjusting to a circumscribed life with few possibilities of medical intervention, those in the latter group emphasise medical science’s ‘potential for mastery over heart disease, through prevention and treatment’ (425). However, in literary fiction at least, this progress of medical science and the attendant rise in survival rates do not seem to have resulted in patients who are any less troubled. Rather, Oldfield and Jones show that the increasing medicalisation and bio-mechanisation of modern medicine in late twentieth and early twenty-first centuries has gone hand in hand with existential concerns and a tangible sense of alienation vis-à-vis medical practice. An interesting question is whether these temporal shifts in the cultural undertones of heart disease are detectable among doctors who experienced cardiac illness. I will present three doctors’ narratives, the first two taken from written illness narratives anthologised in 1952 and 1987 respectively, and the most recent published in December 2015 on a website managed by Taylor Healthcare.

## Three narratives of cardiac illness

### Max Pinner—The sick body objectified

Travelling by night train through North America in the 1930s, the pulmonologist Max Pinner was woken by a new but familiar pain, ‘retrosternal, radiating down along the left arm into the fingertips of my hand’ (1952, 18). He immediately recognised it as angina pectoris. ‘I knew that first moment with the certainty that goes beyond intellectual knowledge, that this was the beginning of a new epoch in my life’ (18). When the angina returned a few days later, it brought Pinner up short, but he still refused to seek medical attention. He was a doctor himself, after all, and thus summarised the anamnesis from his biomedical perspective. ‘The total indictment: hereditary stigmata of vascular disease; history of rheumatic fever; evidence of slowly progressing vascular disease during the last few years’ (20). It was not until six months after the first symptoms that Pinner finally arranged an appointment with a physician (and good friend), who dismissed his symptoms as vasospasm due to the typical mental strain of being a doctor. ‘Did I not know that I was a high-strung person, that I worked and worried too much, that I did not relax properly?’ (20).

Yet to change personality and professional lifestyle was neither easy nor even an option for Pinner. Instead, his strategy was denial. ‘I did not want to have heart disease … and if I had it, it had to be concealed, it could not be permitted to interfere with my work’ (21). As the years passed and his symptoms gradually grew more serious, he consulted several colleagues. Every time he was struck by the feeling that he was less thoroughly cared for than if he had been a ‘regular’ patient. ‘Because I am a physician myself, not more than one or two of my physicians were able … to avoid discussing “my case” with me as if I had been called into consultation. They failed to give definite orders and advice and tended to say, explicitly or implicitly, “You know what to do!”’ (26). In order to protect both him and them from the stigma of disease and the difficulty of the situation, the doctors he consulted did their utmost to hit upon more trivial explanations to his symptoms: vasospasm instead of coronary sclerosis; asthma instead of cardiac failure. And since he did not want to be sick, he played along, trying to persuade himself that his doctors were correct. ‘During the next two years, I often wondered whether my physicians were more successful in fooling me, off and on, or whether I was more successful in fooling them—or at times myself’ (28). Slowly, however, as his symptoms increased in severity, his vague sense of personal responsibility crystallised into self-reproach, and ‘Responsibility—failure—guilt—shame’ (23) played in an endless causal loop in his mind.

Due to his professional background as a doctor, he was denied (and denied himself) access to the role of the patient. All things considered, Pinner did not regard himself as a patient. He barely regarded himself as a doctor with cardiac illness. Instead, in his own eyes he was, first and foremost, a doctor attached to a diseased body. For Pinner, there was no bridging the mental gulf that separated his biomedical understanding of disease from his individual experience of illness. Rather, he did his best to retain his accustomed professional persona as long as possible.

In Pinner’s literary testimony, guilt and frustration are the central themes. His text is a medical case report by a doctor about his own ailing self, where the possibility of being the patient is effectively ruled out by his professional identity.

### Robert Seaver—A testimony to role confusion

The gastroenterologist Robert Seaver had for several years treated himself with antacids for suspected oesophageal reflux when, in December 1982, ‘bands of steel suddenly encircled my chest’ (Seaver 1987, 30). At accident and emergency (A & E), an ECG revealed a heart attack, and a subsequent angiography showed multi-vessel coronary artery disease. Seaver was immediately enrolled and then transferred to a cardiac surgical unit at a neighbouring hospital where he underwent successful bypass surgery. Yet even though every step of his care went according to plan, for Seaver the experience was far from uncomplicated. His medical identity as a doctor proved difficult to combine with the new position he was assigned as a patient. Seaver’s sense of role confusion was considerable: ‘I was being asked to play the game—and I had the misfortune to know all the rules’ (32). In this ‘game’ the dilemmas were multiple, as were the signs of role discrepancy: health-care professionals who did not know whether to address him as a patient or a colleague; curious staff members who failed to respect his integrity as a patient; patients who left his medical practice because he had proved himself fallible, and thus ‘no longer to be completely trusted’ (33).

One practical issue was the lack of confidentiality. In Seaver’s words, his medical records were an open book, and ‘half the people in the hospital and a significant number of my patients and friends knew the anatomy of my coronary circulation better than I’ (32). Yet the dilemmas were also existential in nature. His illness, an insistent reminder of his own vulnerability, the emotional armour he had built up over years of doctoring started to crumble. ‘I had, I now realized with awful clarity, come to regard myself as having entered into a pact with God’ (31). The heightened awareness of his own mortality continued to haunt him long after he resumed his medical practice. Cardiopulmonary resuscitation in A & E, pronouncing a death in a nursing home, attending medical meetings and conferences on heart disease: all these were duties that previously were part of a routine but were now a series of shattering experiences. As the distance between the professional and personal perspectives on medical practice narrowed, his own inescapable fate was daily reflected in the eyes of his patients. ‘What bored and tired doctor with a black ballpoint pen would all to soon be signing “my” death certificate?’ (37) he asked himself.

Role confusion is the dominant theme, as Seaver casts about for a way to switch between the mental configurations of doctor and patient. In what is perhaps the key passage, Seaver points out that ‘It is a doctor’s job to search diligently for the worst. The patient hopes eternally for the best. When they are the same person, the conflict becomes extremely difficult (perhaps impossible) to reconcile’ (37).

### John P. Mulligan—From the patient’s perspective

In early 2015, the emergency medicine physician John P. Mulligan experienced a ‘progressively worsening shortness of breath’ (2015). When climbing the stairs became strenuous, he contacted his primary care physician for an appointment in three weeks’ time. In the interim, Mulligan was sent for an X-ray CT of his chest, which disclosed ‘a 6.8 cm thoracic aneurysm’ stretching the aortic root and bicuspid valve to the point of heart failure. This was when, as he noted himself, ‘the tables turn [ed]’, and with surgery his only option, Mulligan realised that from now on he was first and foremost a patient. From this new viewpoint a previously neglected understanding dawned on him: just how emotionally disparate the doctor’s objective and the patient’s subjective understanding of health and illness truly are. Despite his familiarity with managing life-and-death situations on a daily basis, Mulligan found himself ‘woefully ill-prepared’ for the emotions that unfolded when that life was his own. Under the heading, ‘Good-Bye control, Hello Anxiety’, he described how fear and worry, rather than the physical constraints, were the most burdensome symptoms during his illness. After consulting a friend and former colleague, Mulligan was transferred to ‘a prestigious institution on the East Coast’ and scheduled for aortic valve repair surgery. At this top surgical unit, he was impeccably taken care of: ‘Every day there was a plan that was shared with me and I knew exactly what to expect.’

In contrast to Pinner and Seaver, Mulligan’s professional background as a doctor did not seem to have been much of an issue for the staff who took care of him. The ‘medical drama’ proceeded without complications, tick boxes carefully regulated the care he received, and Mulligan felt confident about his own position in the ‘fast and efficient’ health-care machinery. Still, there was something important missing. As Mulligan remarked, ‘no one ever asked the very basic question—how was I doing? How was my family, what about my work, what were my expectations and how was I coping with possible bad outcomes?’ Personal interaction—neither as a patient nor as a doctor but as an individual—was something Mulligan requested but failed to receive. The hospital service he lacked most had little to do with modern medicine: poor Wi-Fi meant he was unable to say a proper goodbye to his wife and four kids on Skype before being taken away for surgery.

The central theme of Mulligan’s account is whole-person care; the lesson learnt, ‘that as hospitals and providers strive to create institutions that are operationally excellent, we may still miss the mark if we do not know what is really important to the patient.’ Mulligan wanted to be taken notice of as a unique individual, with emotional needs detectable only if the medical staff were to shed the idea that care is a bureaucratic tick-box exercise.

## Discussion

‘The good physician treats the disease; the great physician treats the patient who has the disease’ (Bean and Bean 1961). As this famous aphorism from Sir William Osler (1849–1919) suggests, one essential feature of the medical encounter between doctor and patient is their divergent perspectives on the same central issue of health and illness. There is a dichotomisation between the scientifically observed signs of disease and the socially lived experience of illness (Charon 2006). Doctors and patients see different things, and, as probably all medical practitioners can attest, successful treatment, from the bio-scientific viewpoint, does not always result in a satisfied patient. However, when the patient is also medically qualified, this principal dualism of medical practice is contested, and the expected medical drama risks disorganisation. Here, the complex set of considerations that guide doctors when they become ill are exemplified in the testimonies of Max Pinner, Robert Seaver, and John P. Mulligan. This is not the first analysis to adopt an interpretative approach to written illness narratives by doctor-patients (see, for example, Hahn 1985; Hull 2013; Tischler 2015); however, while earlier articles have focused on narratives from one specific era, the present study has an added historical dimension. From such a temporally comparative approach, it is evident that the experience of becoming a patient while simultaneously being a doctor is a challenge that has changed over time.

It should be stressed that the three narratives in question share many similarities. All three doctors reflect, more or less explicitly, on the essential dualism of medicine. As their identities as doctor and patient merge, they describe the bewildering experience of being threatened by an event that is both unfamiliar *and* simultaneously part of a well-known professional routine. All three also raise the dilemma of what Goffman describes as the coalescence of the front and back regions.^4^ Their awareness of the back region—knowing what staff are thinking about their symptoms and possible complications of medical intervention—is portrayed both as a resource and a menace. Seaver spells it out most explicitly: ‘Foreknowledge is the scourge of the physician-patient. Within moments of the onset of my symptoms, I knew what was to become of me if I survived’ (1987, 29). Given the dramaturgical disorganisation, and in line with the findings of questionnaire- and interview-based studies of deviant help-seeking behaviour among sick doctors, all three doctors also made use of alternative pathways to medical care, including self-medication and informal consultations with friends and colleagues when their symptoms first appeared (Kay et al. 2008). In that sense, to use the medical anthropologists McKevitt and Morgan’s terminology (1997), all three doctors became anomalous patients, since they were ‘at once insiders and outsiders creating ambiguity: the union in one body of two apparent opposites.’

More interesting than these similarities, however, are the differences between the three narratives. As illustrated in Table 1, these differences fall into three categories: (*a*) each doctor’s relationship to professional health care; (*b*) each doctor’s relationship to his own sick body; and (*c*) the overarching theme of each doctor’s narrative. Thus Pinner, even as he suffers from severe anginal pain and shortness of breath due to congestive heart failure, is not allowed or willing to capitulate his professional identity as a doctor. The maxim ‘once a doctor, always a doctor’ is as good as dogma, and he embraces a dualistic approach of almost Cartesian scope with body and mind different entities, and while he admits in objectifying terms the bodily signs of illness, his professional self-image as a doctor remains intact. The difference to Mulligan is striking. He immediately turns into a patient as he enters the surgical unit where his heart valve is to be replaced. Though aware of his professional background, the staff caring for him never deviate from their routines. As their duties are regulated by routines and tick boxes, their patients’ professional identity is unimportant. Compared to Pinner, Mulligan also relates to his own body very differently. His body is himself, part of an indivisible entity, and subjectification rather than objectification is what characterises his relationship to his physical ailment.

**Table 1:** Key differences between the three narratives

	Max Pinner (1952)	Robert Seaver (1987)	John P. Mulligan (2015)
Relationship to health care	Primarily a doctor	Ambivalent roles	Primarily a patient
Relationship to own sick body	Objectification	Complicated	Subjectification
Overarching theme	Frustration	Confusion	Whole-person understanding

The experience described by Seaver in the 1980s is more complex. Like Mulligan, Seaver’s narrative dwells on his personal experience more than on medical events. He is his sick body. At the same time, in the eyes of the staff caring for him, he is first and foremost an ill doctor. In a balancing act between two seemingly irreconcilable identities, he strives to be a model patient and yet clings to his professional identity. The result is ambivalence and confusion, not just as regards Seaver’s own relationship to health care but also in the staff’s relationship with him as a patient. Seaver never becomes a patient on the same terms as Mulligan, since he is bound to a medical identity he cannot escape, even if occasionally he would have preferred to.

When applying Goffman’s theory of social interaction to the three narratives, the role discrepancy is particularly obvious in Pinner’s and Seaver’s accounts. However, a closer look reveals that their role discrepancy is not, in fact, identical. While Seaver is clearly perplexed by what role to adhere to and which script to follow, Pinner rather expresses a sense of frustration, as his accustomed and natural doctor identity proves dysfunctional when seeking medical advice for himself. In that sense, the role confusion inherent in dramaturgical disorganisation is far more salient in Seaver’s narrative than in Pinner’s. Mulligan, on the other hand, never attempts to challenge the role assigned to him after he is admitted to hospital: for him, the impact of illness and becoming a patient is primarily challenging on a private level, while it does not seem to threaten his professional identity to the same extent as for Pinner and Seaver.

Three cases are a precarious foundation for general conclusions, so it should be stressed that rather than to prove causality, the purpose of this analysis is first and foremost to illustrate an experience and nuance our understanding of the sick doctor’s plight. Dissecting the testimonies of Pinner, Seaver and Mulligan can modify the assumption that when doctors become patients, they all have the same experiences. That said, I broaden the analysis to include other narratives by other doctor-patients, and it appears that each of the three testimonies has more in common with other narratives of a similar date than with one another. For example, Seaver’s profound role confusion echoes Oliver Sack’s in *A Leg to Stand On* (1984), or the descriptions in Fitzhugh Mullan’s *Vital Signs: A Young Doctor’s Struggle with Cancer* (1983). Mulligan’s focus on how illness affects his personal rather than professional self calls to mind Paul Kalanithi’s acclaimed testimony *When Breath Becomes Air* (2016). And Pinner’s fixed doctor identity is not so very different to the one described by Mikhail Bulgakov in his semi-fictional short story, “Morphine” (1927) (Bulgakov 1975; Wistrand 2017). It thus seems reasonable to argue that doctors’ experiences of personal illness and treatment has changed over time, making it a less frustrating and confusing event for contemporary doctors than it was in decades earlier.

A central question is whether the observed differences are primarily caused by changes in medical practice or in society at large. The most likely answer is both. Since Pinner was writing in the 1940s, medical science has advanced at an amazing speed. Surgical and pharmacological interventions have improved, and medical algorithms regulate healthcare in a more standardised way than ever before. Where heart disease for Pinner is a fateful verdict in which death could at best be delayed by various medications of dubious effect, for Seaver and Mulligan, it is rather an acute threat, possible to fend off by surgical and pharmacological interventions. But equally important, the role identity of the physician has changed over time. Doctoring has become a job of work rather than an all-embracing lifestyle. The hierarchical construction of health care has been replaced by a system of cooperation and mutual responsiveness among all members of the team, including the patient. As a consequence, the role identity of the doctor has become more flexible, more likely to combine with a parallel, private identity with all its deficiencies and shortcomings. It also seems likely that greater access to medical information has made healthcare professionals in general more accustomed to caring for patients with preconceived ideas and opinions about the medical treatment on offer. In that sense, being a doctor-patient today bears more resemblance to the experience of ‘conventional’ patients than it did a few decades ago—changes might explain why Mulligan’s account of cardiac illness focuses far less on role discrepancy than Pinner and Seaver do (Ingstad and Moe Christie 1996).

Still, it is evident that Mulligan is not altogether satisfied. Rather than being treated as a pawn in a medical drama, he hopes to be seen as a unique individual, without the template of the patient-character obscuring his personal needs and emotions. In that sense, Mulligan’s narrative corresponds to Oldfield and Jones’s findings that alongside the increasing bio-mechanisation of cardiology (as in most medical disciplines) there is a growing sense of alienation from medical practice. In his personal resentment, Mulligan pinpoints one of the major challenges of contemporary medicine: how to combine care built on generalised algorithms and technical devices with medical practice where there is time, space, and genuine interest in each individual?^5^

It is right to ask whether the fact that all three doctors in question are men influences the experiences they describe. In the anthology in which Seaver’s narrative appears, there is also an account by Judith Alexander Brice, a psychiatrist suffering from a severe bout of ulcerative colitis. Crucially, Brice’s narrative dwells just as much as Seaver’s (if not more) on the sense of role confusion arising from the experienced dilemma of two conflicting identities:

During these hospitalizations I found it very difficult to switch gears. As a doctor I was functioning in a profession of strength, of intellectual activity, of control, and of independence. These qualities did not easily mesh with those of a passive, compliant patient. One minute I was the psychiatric consultant, heading up case conferences, seeing private patients, and supervising residents. … The next moment I was in the hospital with everyone from maintenance crew and janitorial staff to medical staff feeling entitled to enter my room without knocking. (Brice 1987, 175)

In other words, in the medical world Goffman’s principle of role discrepancy seems applicable to men and women alike.

## Conclusion

The purpose of this article is to highlight the potential difficulties of doctors becoming patients, and how these difficulties have changed over time. A sick doctor has to set aside his or her professional role both physically and symbolically, when forced to become a patient, and the search for a bridge between the two identities of doctor and patient might turn out to be a difficult one. Drawing on Goffman’s analogy of medicine as a drama, Ingstad and Moe Christie have noted how the role dilemmas that face doctors if they become seriously ill ‘have no predetermined script, but have to be solved along the way’ (2001, 209). Similar to the multitude of individual experiences described by doctor-patients in their pathographical testimonies, there is probably not one but several possible solutions to how to re-establish a functioning role division between sick doctors and their care providers. Still, as a common principle in the renegotiation of any sick doctor’s medical drama, one should bear in mind what the sociologist Arthur Frank said about medical workers always ‘playing parts in the drama of the ill person’s plight, and that how they play their parts shapes this drama just as consequentially as the disease—the cellular pathology—shapes it’ (2007, 380). As a complement to questionnaire- and interview-based studies, doctors’ written illness narratives promise to help increase health-care professionals’ awareness of the underlying principles of this intricate drama of the sick doctor.
